# Advancing Smart Textiles: Structural Evolution of Knitted Piezoresistive Strain Sensors for Enabling Precise Motion Capture

**DOI:** 10.3390/polym15193936

**Published:** 2023-09-29

**Authors:** Mareen N. Warncke, Carola H. Böhmer, Carmen Sachse, Susanne Fischer, Eric Häntzsche, Andreas Nocke, Johannes Mersch, Chokri Cherif

**Affiliations:** 1 Institute of Textile Machinery and High Performance Material Technology, TUD Dresden University of Technology, 01069 Dresden, Germany; 2 Centre for Tactile Internet with Human-in-the-Loop (CeTI), TUD Dresden University of Technology, 01069 Dresden, Germany

**Keywords:** wearables, smart textiles, textile strain sensor, motion monitoring, medical applications

## Abstract

Recently, there has been remarkable progress in the development of smart textiles, especially knitted strain sensors, to achieve reliable sensor signals. Stable and reliable electro-mechanical properties of sensors are essential for using textile-based sensors in medical applications. However, the challenges associated with significant hysteresis and low gauge factor (GF) values remain for using strain sensors for motion capture. To evaluate these issues, a comprehensive investigation of the cyclic electro-mechanical properties of weft-knitted strain sensors was conducted in the present study to develop a drift-free elastic strain sensor with a robust sensor signal for motion capture for medical devices. Several variables are considered in the study, including the variation of the basic knit pattern, the incorporation of the electrically conductive yarn, and the size of the strain sensor. The effectiveness and feasibility of the developed knitted strain sensors are demonstrated through an experimental evaluation, by determining the gauge factor, its nonlinearity, hysteresis, and drift. The developed knitted piezoresistive strain sensors have a GF of 2.4, a calculated drift of 50%, 12.5% hysteresis, and 0.3% nonlinearity in parts.

## 1. Introduction

Smart textiles offer tremendous potential in healthcare, providing solutions for monitoring vital parameters, facilitating rehabilitation, and improving overall well-being. Various studies have explored the integration of smart textiles into clothing and wearable devices for continuous health monitoring. These devices have demonstrated the ability to collect physiological data, track movement, and provide personalized feedback to patients and healthcare professionals [1,2,3,4,5].

This research work is related to applications in medical smart textiles, for example, motion analysis for rehabilitation applications, or it serves as a signal for electrodes that are intended to stimulate specific muscle groups, e.g., to help people with peroneal nerve paralysis to walk or to train the muscle groups specifically through stimulation [6]. Pressure sensors are one way of analyzing movement, as shown in studies [7,8,9]. However, all existing solutions for motion detection have been implemented via pressure sensors, which were attached to a garment afterwards and not integrally, processed in a manufacturing step and could not be considered as the knitted piezoresistive strain sensor. Due to the high flexibility and elasticity of knitted structures, they are particularly suitable for applications close to the body for medical smart textiles [10]. In [11], a yarn-shaped pressure sensor was previously developed, which was integrated into a knitted fabric to measure the pulse of a human being. It was shown that knitted piezoresistive pressure sensors were a suitable solution in medical applications with low hysteresis. However, this study did not investigate piezoresistive strain sensors for motion capture.

Certainly, some researchers have previously worked with strain sensors suitable for motion capture. Therefore, one option to record the movement is through strain sensors [12,13], which work according to the piezoresistive effect [14,15]. For example, using piezoresistive strain sensors, placed on the joints, the change in the electrical resistance of the piezoresistive strain sensor during mechanical deformation [16,17] can be used to infer joint bending and, thus, the wearer’s movement [2,18,19]. However, in addition to the position of the sensors, the conductive material, structural implementation, and design are also important for sensor functionality. Typical fiber-based conductive materials and their influence on the stability of the sensor signal are analyzed in [17,20,21]. It was found that metalized yarns were suitable in principle; however, the elongation of the base material played a significant role in the sensor signal of a piezoresistive strain sensor and that less stretchable steel yarns were not suitable in contrast to silver-plated polyamide yarns. In [21,22,23,24], for example, the performances of different knitted piezoresistive strain sensors were investigated. The authors explored the optimization of design parameters to achieve enhanced sensitivity and accuracy in strain measurements. The findings demonstrate that the selection of the conductive area and the design of the sensor profile have a significant influence on the performance of knitted piezoresistive strain sensors and are crucial aspects in the development of such sensors. The basic textile structure of the sensors was incorporated exclusively into a mock rib knit (similar to a right–right pattern). [25] focused on addressing the challenge of hysteresis, which can negatively impact the accuracy and reliability of strain measurements. Through the utilization of specialized knitting techniques and the careful selection of materials, the authors successfully design piezoresistive strain sensors that exhibited minimal hysteresis and provided a linear response to the applied strain. The findings demonstrate the potential of weft-knitted piezoresistive strain sensors as reliable and accurate components in smart textiles, opening up opportunities for their integration into various wearables and healthcare applications.

In addition to the essential strain sensor development, various research approaches exist for capturing motion. Weft-knitted piezoresistive strain sensors are already used in various smart textiles for different applications; some of them can be transferred to medical applications. In [26], a smart glove was developed to measure the joint angles of interphalangeal joints. It was shown here that knitted sensors would be suitable for motion capture. The finger movement with a knitted piezoresistive strain sensor was also developed and integrated into a wearable system in [27]. The new piezoresistive strain sensor has the potential to be used for medical smart textiles and the findings assist in performing additional research.

In addition to knitted piezoresistive strain sensors, other material approaches to motion sensing have been investigated. In [28], conductive elastomer sensors were composed of graphite and silicon rubber and, in [29], carbon-filled elastic rubber were coated onto a textile. Study [30] deposited an elastic strain sensor composed of silver–zinc oxide onto a knitted fabric, creating an electrically conductive sensor surface. These variants are also suitable in principle for joint angle measurements. These alternative material approaches are compared in [16], and the advantages and disadvantages are discussed. It became clear that the coating variants still had significant weaknesses and must first be eliminated for medical applications.

Due to the great advantages of knitted piezoresistive strain sensors, this approach is focused on in this paper.

The studies discussed the materials, designs, and the electro-mechanical behavior of knitted piezoresistive strain sensors. The development of knitted strain sensors with low hysteresis is challenging. The main findings from the literature are:

- Steel-based conductive yarns are less suitable for strain sensors due to their low stretchability. Therefore, it is recommended to use silver-coated nylon yarns instead [17,20,21,24].
- The knitted pattern, in which the sensor yarns are integrated, should possess no plasticity or viscosity. Otherwise, the sensor’s response is not elastic, leading to hysteresis and drift effects [20,21].
- In the studies, mainly a right–right pattern is used for the basic knit. The influence of other knitted pattern on the sensor performance is not investigated. Moreover, it is therefore addressed in this study since it can be assumed that the pattern influences the sensor quality [20,22,23].
- The knitted pattern in [25] consists of only one elastic yarn, and the influence of inserting the strain sensor in stitch wale or stitch course directions is not analyzed. For this reason, the knitting direction of the sensors is considered as a criterion for selecting the correct sensor design.
- Only a few of the studies presented here investigate the electro-mechanical properties of strain sensors under multi-cyclic mechanical deformation and thereby verify their suitability as strain sensors for motion capture a.o. in medical applications.

Therefore, in this research, the principle of knitted piezoresistive strain sensors for motion capture in medical applications is explored. As shown by the presented studies, knitted textiles have unique properties, such as flexibility and stretchability, making them ideal for wearable applications. Knitted textiles additionally have the potential to create a weft-knitted piezoresistive strain sensor with low drift, hysteresis and nonlinearity, and high elongation and gauge factor (GF).

## 2. Materials and Methods

**Yarn materials.** In Table 1, the yarn materials and their characteristics are presented. Cellulose-based fibers TENCEL Lyocell (TL) and an elastic filament yarn (EY) formed the basic pattern. The material is particularly suitable for use in medical devices worn close to the body as TL is gentle on the skin and excellent in terms of moisture buffering and sensorial comfort [31]. Elastic yarn was also used to make the knit-ted fabric even more flexible and close-fitting for optimum skin contact for direct motion capture for therapeutic purposes. The SY was used to manufacture the textile-based piezoresistive strain sensors.

**Manufacturing method.** A flatbed knitting machine ADF 530-32 BW knit and wear from H. Stoll AG & Co. KG (Reutlingen, Germany) with the machine unit E7.2 was used to manufacture the piezoresistive strain sensors for enabling motion capture for medical and therapeutic applications. The knitted structures produced using the flatbed knitting machine had different textile parameters. These parameters are listed in Table 2. While producing the strain sensors, the electrically conductive yarns ere fully plated as a mesh with the Dorlastan, depending on the knitting direction in the stitch course or wale direction. Thereby, each sensor mesh consisted of electrically conductive yarn.

The nomenclature of the piezoresistive sensors used the following coding: Knitting direction_Pattern_Strain sensor widthxStrain sensor length, such as W_RL_1 × 200. Table 3 shows examples of the variants of the different knitted patterns used for the study.

**Measurement method.** To determine the resistance change during the cyclic mechanical elongation of the piezoresistive strain sensor, a four-wire-resistance measurement according to DIN EN 16,812 [32] was used. The four-wire-resistance measuring station consisted of a precision multimeter Keithley DAQ 6510 (Tektronix Inc., Beaverton, OR, USA) to determine the measured values, and a computer for data storage and processing. To stretch the specimen, the textile was clamped in the tensile testing machine Z2.5 (ZwickRoell GmbH & Co. KG, Ulm, Germany). The test regime is listed in Table 4.

**Figure 1:**
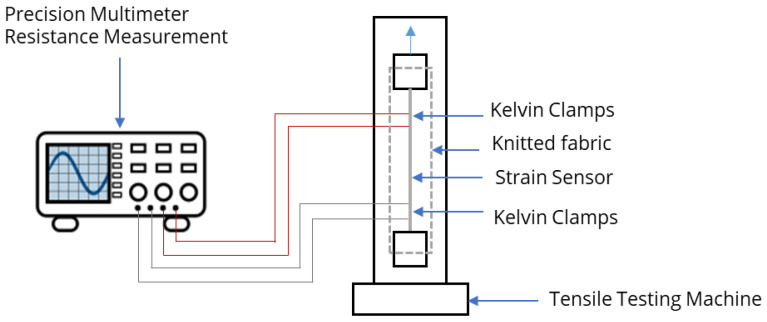
Schematic measurement setup with precision multimeter, tensile testing machine, and fixed piezoresistive strain sensor in four Kelvin clamps.

To measure the piezoresistive strain sensor, it was fixed via four crocodile clips with a distance of 150 mm apart (Figure 1).

**Figure 2:**
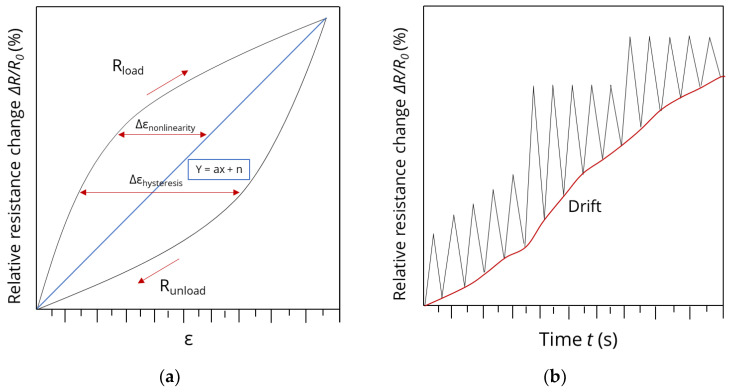
Illustration of determination of the nonlinearity: hysteresis consisting of the relative resistance change in dependence of the elongation (**a**) and baseline drift over time (**b**).

**Analysis of electro-mechanical properties.** In order to evaluate the sensor quality of differently knitted piezoresistive strain sensors, the electro-mechanical properties were determined. The gauge factor (GF), its nonlinearity, hysteresis, and drift were calculated to analyze the cyclic sensor behavior. The software tool MATLAB was used for the analysis. The nonlinearity was determined as the difference between the linear fit line Y and real averaged result of R_load_ (Figure 2a), and was calculated with Equation (1). To calculate the GF later on, the intersection of the Y-scale *n* was set to zero, as *n* is discussed for the drift.

(1) $${Y=ax+n\;}_{\;}\;n{}^{!};=0$$

The GF was derived from the slope of the linear fit. Equation (2) presents the formulation for determining the gauge factor, where Δ*R* denotes the variation in resistance Δ*R* = *R*_load_ − *R*_0_, *R*_0_ represents the initial resistance of the unloaded strain sensor, and Δ*ε* signifies the strain alteration within the linear domain of the curve.

(2) $${GF=\frac{\left(\frac{∆R}{R_{0}}\right)}{∆\varepsilon }}_{\;}$$

The hysteresis values were determined along the strain axis (horizontal), called strain hysteresis. The hysteresis strain was defined as the maximum difference between the loading and unloading strains (see Equation (3)) [33]:

(3) $${H_{\varepsilon }=\max\;\left|\left(R_{load}-R_{unload}\right)\right|}_{\;}$$

In addition to the hysteresis as a measure of the sensor quality, the drift was determined too. The sensor drift was calculated by determining the strain at 0% strain for every sample, averaging these values and determining the maximum deviation from *R*_0_.

## 3. Results

### 3.1. Analysis of Various Piezoresistive Strain Sensors in Stitch Wale and Stitch Course Directions

**Figure 3:**
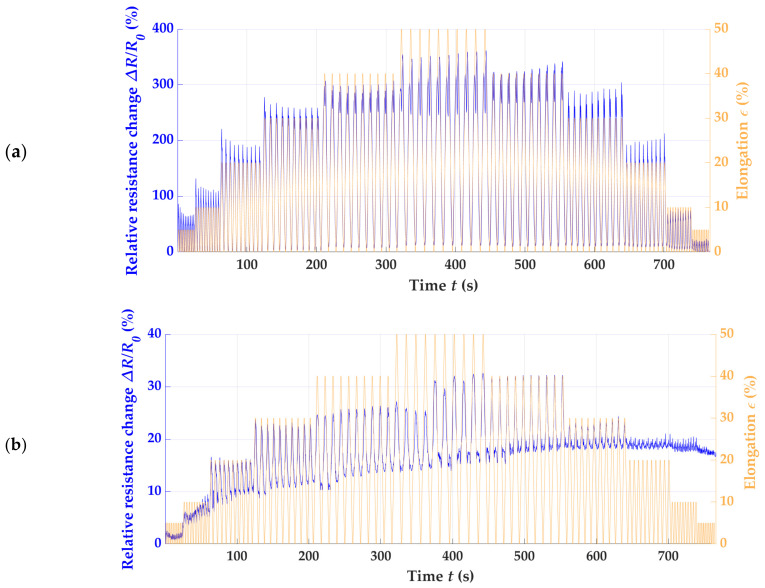
The elongation (in orange) and relative resistance change (in blue) over time of (**a**) W_RL_2 × 200 and (**b**) C_RL_2 × 200.

To comprehensively analyze various piezoresistive strain sensors, the initial phase involved assessing the electrical resistance in relation to strain and time. This assessment allowed for qualitative distinctions to be made. To illustrate the range of outcomes, we focused on the W_RL_2 × 200, C_RL_2 × 200, W_RR_2 × 200, W_RRI_2 × 200, and W_LL_2 × 200 variants as representative examples, since these patterns offered the best overview of the differences between the sensor designs. The first example was the knit knitting direction and the differences between the stitch wale and stitch course directions. The results are shown in Figure 3a,b.

**Figure 4:**
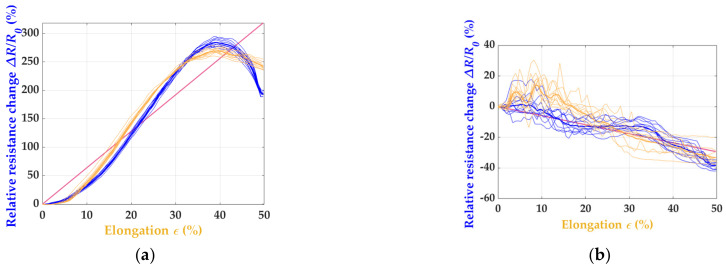
The correlation between relative resistance change (in blue) and elongation (in orange) of (**a**) W_RL_2 × 200 and (**b**) C_RL_2 × 200.

The sensor oriented in stitch wale direction showed a significantly better correlation between the strain limits and relative resistance change. From a purely qualitative point of view, the sensor’s performance was promising. The quantification of the sensors’ properties are considered and discussed later. In contrast to W_RL_2 × 200, the electrical resistance and strain did not correlate as significantly for the sensors fabricated in the stitch course direction, and the sensor’s apparent drift and hysteresis over time can be seen in Figure 3b. Based on this preliminary investigation, the knitting direction had a decisive influence on the sensor metrics, such as the nonlinearity, GF, drift, and hysteresis. The results suggest that the knit pattern in the stitch course direction, as shown in Figure 3b, is less suitable for a piezoresistive strain sensor for motion capture. For a better understanding, the actual values of both sensor variants were determined, as explained theoretically in Figure 2a, and shown graphically in Figure 4. From the linearity line (purple), it can be seen in (b) that the sensor performance deviates strongly, and high irregular hysteresis and nonlinearity can be recognized. The results already indicated in Figure 3 are thus confirmed. For the W_RL_2 × 200 variant (a), low hysteresis and nonlinearity can be seen up to a proof stress of 30%, which indicates a stable sensor performance up to this strain limit.

Therefore, the following step was to examine and compare the different pattern variants only in the stitch direction.

### 3.2. Analysis of Various Patterns and their Piezoresistive Strain Sensors

**Figure 5:**
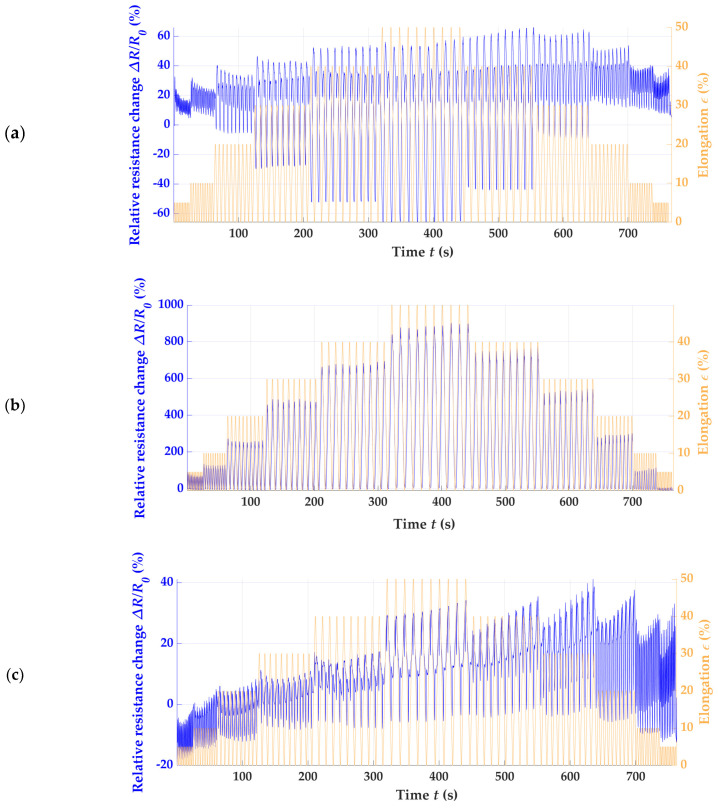
The elongation (in orange) and relative resistance change (in blue) over time for (**a**) W_RR_2 × 200, (**b**) W_RRI_2 × 200, and (**c**) W_LL_2 × 200.

The analysis was based on the variants W_nn_2 × 200 (Figure 3a or Figure 5a–c). Figure 3a depicts the data for the sample W_RL_2 × 200 and Figure 5b for the sample W_RRI_2 × 20, again showing the electrical resistance and strain, whereas the sensor signals of (a) W_RR_2 × 200 and (c) W_LL_2 × 200 drifts over the cycles. The best result indicates the W_RRI_2 × 200 pattern. The graphene’s strain and resistance change conform, as seen in (b).

**Figure 6:**
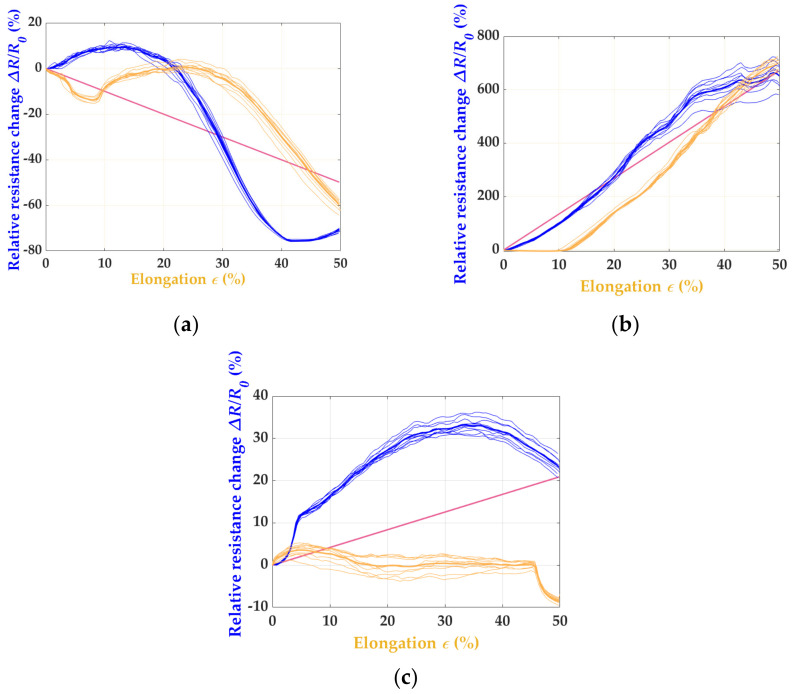
The correlation between the relative resistance change (in blue) and elongation (in orange) of (**a**) W_RR_2 × 200, (**b**) W_RRI_2 × 200, and (**c**) W_LL_2 × 200.

For determining the sensor quality, the correlation of the resistance change and elongation are essential features, as shown in Figure 2a, which shows in the following step to calculate the nonlinearity and hysteresis of the (a) W_RR_2 × 200, (b) W_RRI_2 × 200, and (c) W_LL_2 × 200 patterns in Figure 6. It can be seen that the strain sensor shows hysteresis and nonlinearity up to 15% in (a). From 20%, a decrease in the sensor performance can be seen.

The results suggest that the knit pattern also influences the sensor quality and that RL and RRI might be more suitable for a piezoresistive strain sensor for motion capture.

### 3.3. Analysis of the Nonlinearity, Gauge Factor, Drift, and Hysteresis of Various Patterns and Its Piezoresistive Strain Sensors

**Figure 7:**
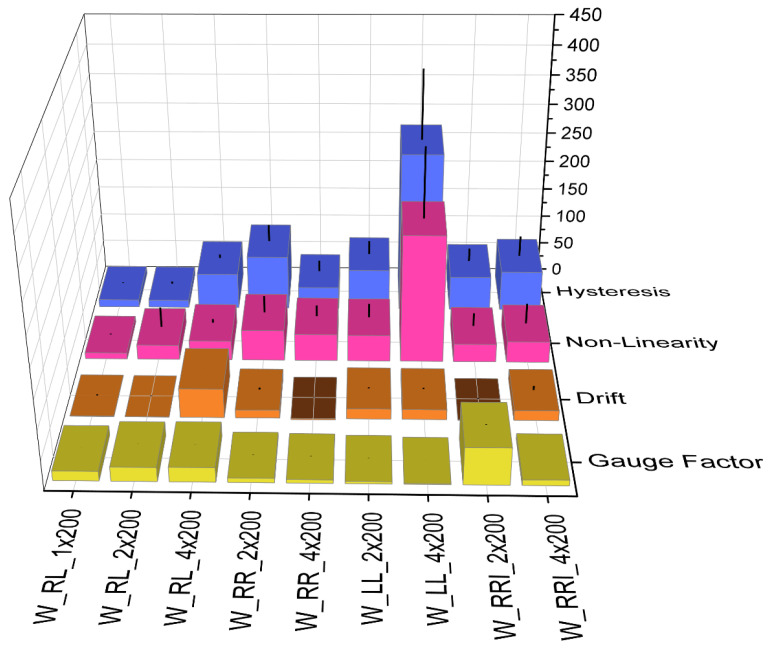
Effect of knitted pattern and the differences between right–left, right–right, right–right–interlock, and left–left patterns and piezoresistive strain sensor width, and the differences between one, two, and four meshes in line on the sensor performance nonlinearity, gauge factor drift, and hysteresis.

Based on these findings, the nonlinearity, GF, drift, and hysteresis are determined in the following step, and the results are presented in the following section. Figure 7 illustrates the effect of the knitted pattern and the differences between right–left, right–right, right–right–interlock, and left–left patterns. The effect of the sensor width and the differences between one, two, and four meshes in a line were also examined.

Upon examining the nonlinearity, it was evident that variant W_LL_4 × 200 exhibited a deviation of over 200%. The same observation applied to the hysteresis. However, a clear correlation between the pattern, sensor width, and the resulting sensor quality could not be determined based on these results. On the other hand, the analysis of the correlation between the strain and electrical resistance in Figure 5 indicates that the variant LL and RR patterns are unsuitable for the intended purpose. The patterns with the best transmission behavior and the lowest drift were W_RL_4 × 400, W_RL_2 × 200, W_RL_1 × 200, W_RRI_4 × 200, and W_RRI_2 × 200. The correlation between the strain and resistance over time for the best and worst variants can be seen, for example, in Figure 3a and Figure 5b,c. These samples differed in the RL and RRI patterns and sensor width. The W_RRI_4 × 200 variant had a GF of 0.9 and a drift of 17%. The W_RRI_2 × 200 variant had the highest GF of 6.3 and the lowest drift of 0.15%. Despite having the same pattern and knitting direction, the difference could be attributed to the sensor width, which is discussed further, later. The RL pattern with widths of W_RL_4 × 400 and W_RL_2 × 200 had a GF of 2.4, a drift of 50%, and 0.3% nonlinearity, while the W_RL_1 × 200 variant had a GF of 1.6 and a drift of up to 0.8%. Again, the results differ because of the sensor width rather than the pattern. When considering the sensor width, it can be observed that the variants with four meshes exhibited a nonlinearity of 33.5% and a hysteresis of 61.5% for the RL pattern. For the RRI pattern, the values were in a similar range with a nonlinearity of 36% and a hysteresis of 69%. Reducing the width to two meshes also reduced the nonlinearity to 25% and the hysteresis to 12.5% for the RL pattern. For RRI, the nonlinearity was 31% and the hysteresis was 58%. Despite having a high GF and low drift, the deviations in terms of linearity and hysteresis were more significant for W_RRI_2 × 200 compared to the RL variants. Although W_RL_1 × 200 had a lower GF, it exhibited the lowest nonlinearity at 10.7%.

## 4. Discussion

**Figure 8:**
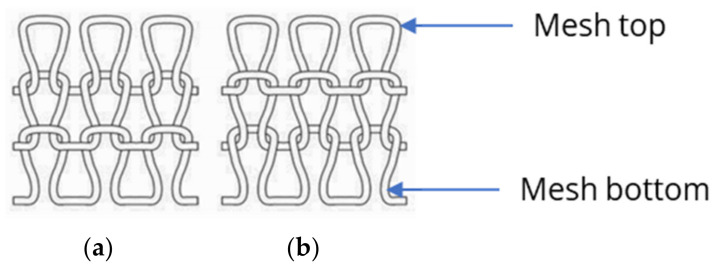
LL pattern with face (**a**) and back (**b**) sides [34].

Based on the preliminary investigation, the knitting direction had a decisive influence on the sensor metrics, and the C direction was unsuitable as a knitting direction for a piezoresistive strain sensor. In addition to the knitting direction, the knit pattern also influenced the sensor quality. The results support the hypothesis that LL and RR patterns are unsuitable and that RL and RRI patterns might be more suitable for a piezoresistive strain sensor for motion capture, as indicated by the correlation between the strain and resistance change in Figure 5. Based on Figure 7, it can be assumed that the LL and RR patterns might be more unsuitable for a piezoresistive strain sensor because the GF is lower than 0.8%. In comparison with all four characteristics, it can be assumed that the LL and RR patterns might be less suitable for a piezoresistive strain sensor with reliable properties. The mesh tops always alternate above or below the mesh bottoms in an LL pattern (Figure 8).

The alternation between the contact points of the mesh tops and bottoms in the sensor might lead to an indifferent change in the resistance when stretched, since the mechanical strain and, thus, the electrical resistance probably have a different effect on the crossing point lying above the mesh than on one lying below it. As the sensor geometry changes due to strain, the connection points within the sensor also vary, resulting in fluctuations in the sensor performance.

**Figure 9:**
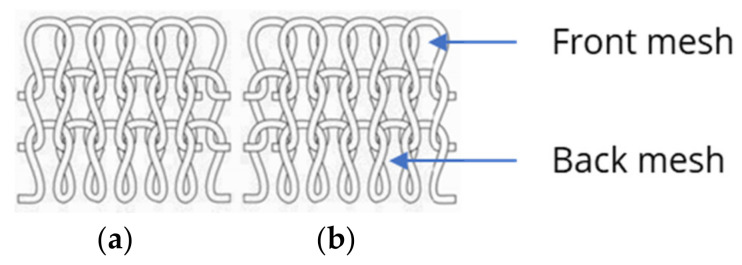
RR pattern with front and back meshes, and face (**a**) and back (**b**) sides [34].

**Figure 10:**
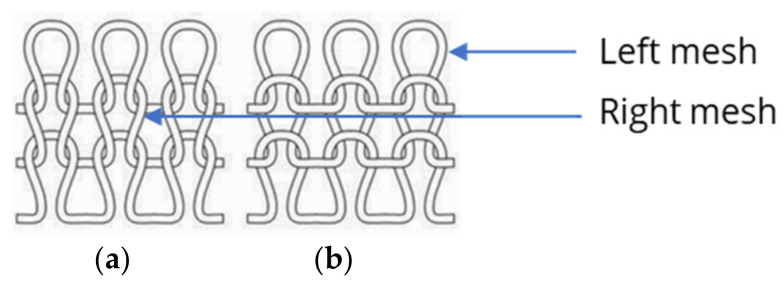
RL pattern showing left and right meshes with face (**a**) and back (**b**) sides [34].

For the RR pattern (see Figure 9), the mesh top and bottom interlock in the same way as for the RL pattern (see Figure 10), with the difference being that the meshes are knitted on two needle beds, and the front mesh is continuously interlocked with the back mesh. In this way, the stitch wales from the front and back needle beds always alternate, resulting in a knit with twice as many meshes as in the RL pattern.

Therefore, it would be expected that an RR pattern would behave in the same way as the RL knit. For both patterns, only right meshes on the right side and left meshes on the left side can be seen. Because the mesh bottom and top always interlock, the sensor has a high degree of stretch and good transmission behavior, especially for the RL knit. However, twice the number of meshes results in a minor change in the sensor geometry and resistance change for the same strain, which leads to the poor GF behavior of the sensor.

**Figure 11:**
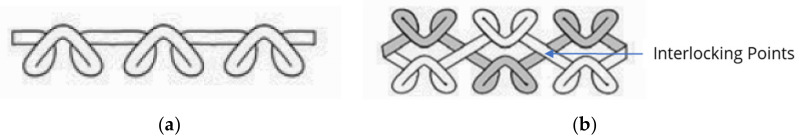
Top views of RL pattern (**a**) and RRI showing the diagonally interlocking points (**b**) [34].

The results show that the two variants, W_RL_1 × 200 and W_RRI_2 × 200, have a reliable sensor performance but show a difference in nonlinearity and hysteresis. The distinction between the two pattern variants occurred due to the interlocking points between the mesh tops and bottoms. For the RRI pattern, the interlocking is always offset by one mesh diagonally (see Figure 11b), resulting in more significant deviations in the electrical resistance within the sensor during stretching in contrast to the RL pattern. For the RL knit, there is only one row of meshes (Figure 11a) and, as already described above, the mesh top and bottom are always in contact without interlocking.

In addition to the interlocking points, the sensor of the RRI pattern consisted of twice as much material as the RL pattern.

The results obtained in this research significantly contribute to the state of the art and tie in directly with the summarized findings in the Introduction. As discussed, the influence of knitted patterns, such as right–left, left–left, or interlocked ones on the sensor’s performance, still needs to be investigated. Nevertheless, in this systematic study, the influence of the different patterns were discussed, and the differences concerning the sensor behavior can be elaborated. Above all, the sensors’ hysteresis was also influenced by the knitted pattern hysteresis since the mechanical stress’s immediate effect was delayed at higher strength limits, depending on the mesh intersection. It can be assumed that residual elongation was still present in the knitted pattern and could not relax entirely before a new elongation cycle began. This was added to the evaluation of the sensor performance and should be systematically investigated in future studies to improve the performance of knitted strain sensors. It was also established that the knitting direction influenced the selection of the correct sensor design. The electro-mechanical properties of the strain sensors under multi-cyclic mechanical deformation and their suitability as strain sensors for motion capture a.o. in medical applications were investigated. It was found that the knitted sensors showed reliable sensor behavior, even under high cyclic loading and would be suitable for long-term use under different stresses.

## 5. Conclusions

In summary, the W_RL_1 × 200 and W_RRI_2 × 200 variants were the piezoresistive strain sensors with the best sensor performance and might be ideal for use in smart textiles for medical applications. For selecting the appropriate knitted sensor, it was necessary to compare all four properties and weigh the parameters that should be considered in each dimension and the ones that could be somewhat neglected. Ultimately, right–left and right–right–interlock patterns were the most suitable knitted structures for a piezoresistive strain sensor, with RRI exhibiting higher nonlinearity and hysteresis than the RL pattern. However, the width of the sensor was more crucial for sensor quality. A sensor width of four meshes resulted in reduced the sensor quality regardless of the pattern, while a width with two meshes for the RRI pattern and width with one mesh for the RL pattern were the best variants considering all four quality criteria. The questions regarding why adding one or two meshes significantly affects the sensor’s performance remain unanswered. Additionally, why the knitting direction caused a sensor drift also remains unsolved in the study. For this reason, further studies are needed to investigate the influences of the sensors’ width and knitting direction. The knowledge about these sensors’ properties is necessary to design and manufacture different piezoresistive strain sensors to meet the requirements of various medical applications. The findings of this study contribute to the ongoing research and development efforts in the field of smart textiles, particularly in the domain of medical applications.

## Figures and Tables

**Table 1:** Yarn materials used for the wearable piezoresistive strain sensors.

Producer	Name	Composition	Linear Density (dtex)	Resistance (Ω·m^−1^)	Nomenclature
Gebr. Otto GmbH + Co. KG	TENCEL Lyocell Std. Ringgarn	TENCEL Lyocell	25	-	TL
Jörg Lederer GmbH Elastic-Garne	Dorlastan 160 DC	Dorlastan/PA 6.6	270	-	EY
Amann & Söhne GmbH & Co. KG	Silver-tech + 150	PA 6.6	220	<300	SY

**Table 2:** Textile parameters of the different patterns.

Knitting Direction	Pattern	Strain Sensor Width (mm)	Strain Sensor Length (mm)	Nomenclature
Stitch wale direction	right–left	1	200	W_RL_1 × 200
Stitch wale direction	right–left	2	200	W_RL_2 × 200
Stitch wale direction	right–left	4	200	W_RL_4 × 200
Stitch wale direction	right–right	2	200	W_RR_2 × 200
Stitch wale direction	right–right	4	200	W_RR_4 × 200
Stitch wale direction	right–right–interlock	2	200	W_RRI_2 × 200
Stitch wale direction	right–right–interlock	4	200	W_RRI_4 × 200
Stitch wale direction	left–left	2	200	W_LL_2 × 200
Stitch wale direction	left–left	4	200	W_LL_4 × 200
Stitch course direction	right–left	200	2	C_RL_2 × 200

**Table 3:** Overview of the variance of the different patterns.

	W_RL_2 × 200	W_RR_2 × 200	W_LL_2 × 200	W_RRI_2 × 200	C_RL_2 × 200
Face side	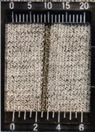	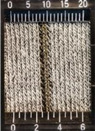	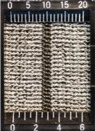	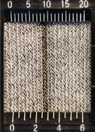	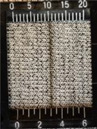
Reverse side	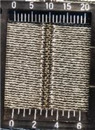	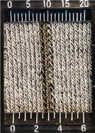	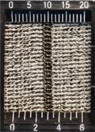	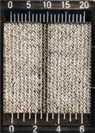	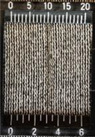

**Table 4:** Parameters of the test regime.

Parameter	
Clamping length	200 mm
Preload	0.5 N
Testing speed	1000 mm/min
Strain in the range from	0% to 5%, 0% to 10%, 0% to 20%, 0% to 30%, 0% to 40%, 0% to 50% and 50% to 0%, 40% to 0%, 30% to 0%, 20% to 0%, 10% to 0%, 5% to 0%,
Release control	elongation limits
No. of cycles	10

## Data Availability

Data available on request due to restrictions, e.g., privacy or ethical. The data presented in this study are available on request from the corresponding author. The data are not publicly available due to privacy.
